# Cannabis Use and Mental Illness: Understanding Circuit Dysfunction Through Preclinical Models

**DOI:** 10.3389/fpsyt.2021.597725

**Published:** 2021-02-05

**Authors:** Bryan W. Jenkins, Jibran Y. Khokhar

**Affiliations:** Department of Biomedical Sciences, Ontario Veterinary College, University of Guelph, Guelph, ON, Canada

**Keywords:** schizophrenia, major depressive disorder, bipolar disorder, cannabis use disorder, oscillations, electrophysiology

## Abstract

Patients with a serious mental illness often use cannabis at higher rates than the general population and are also often diagnosed with cannabis use disorder. Clinical studies reveal a strong association between the psychoactive effects of cannabis and the symptoms of serious mental illnesses. Although some studies purport that cannabis may treat mental illnesses, others have highlighted the negative consequences of use for patients with a mental illness and for otherwise healthy users. As epidemiological and clinical studies are unable to directly infer causality or examine neurobiology through circuit manipulation, preclinical animal models remain a valuable resource for examining the causal effects of cannabis. This is especially true considering the diversity of constituents in the cannabis plant contributing to its effects. In this mini-review, we provide an updated perspective on the preclinical evidence of shared neurobiological mechanisms underpinning the dual diagnosis of cannabis use disorder and a serious mental illness. We present studies of cannabinoid exposure in otherwise healthy rodents, as well as rodent models of schizophrenia, depression, and bipolar disorder, and the resulting impact on electrophysiological indices of neural circuit activity. We propose a consolidated neural circuit-based understanding of the preclinical evidence to generate new hypotheses and identify novel therapeutic targets.

## Introduction

Cannabis is one of the most widely used psychoactive substances worldwide, and patients with serious mental illnesses use cannabis at rates much greater than the general population (1, 2). The lifetime cannabis-use rates for patients with schizophrenia, major depressive disorder, or bipolar disorder are 80, 17, or 24%, respectively, while ~40% of patients with schizophrenia and 20% of patients with major depressive disorder or bipolar disorder are also diagnosed with cannabis use disorder (3–6). A diagnosis of mental illness increases the risk for lifetime cannabis use, while cannabis use, especially use of greater potency cannabis at an earlier age, increases the risk for developing a mental illness (1, 7). Therefore, there is a need for mechanistic investigations that can then be targeted toward developing novel treatment approaches (8, 9). To improve our understanding of cannabis use and serious mental illness, we herein provide an update on the preclinical evidence (Table 1) in support of shared neurobiological mechanisms fundamental to the effects of cannabis and the symptoms of mental illness.

**Table 1:** Summary of cited studies including the rodent model used, cannabinoid and route administered, as well as the aims and outcomes of each study.

**Study**	**Modeled illness**	**Animal model and strain**	**Cannabinoids and dose**	**Administration route**	**Aims**	**Outcomes**
Abush and Akirav (10)	Major depressive disorder	Chronic restraint stress in Sprague Dawley rats	WIN55-212,2, 1.2 mg/kg; AM-251, 0.3 mg/kg	i.p.	Examine limbic glucocorticoid receptor and synaptic plasticity changes after chronic stress and/or cannabinoid exposure	Chronic cannabinoid exposure prevented stress-induced impairments in plasticity, and was CB1R-dependent
Aguilar et al. (11)	Schizophrenia	Subchronic PCP-treated Sprague Dawley rats	URB597 (FAAH inhibitor), 0.3 mg/kg	i.p.	Examine whether increasing anandamide (via URB597) will reverse aberrant VTA DA neuronal activity	PCP-treated rats exhibit enhanced baseline VTA DA neuronal population activity compared to controls; URB597 administration reversed this effect
Aguilar et al. (12)	Schizophrenia	Subchronic PCP-treated Sprague Dawley rats	THC, 1 mg/kg; URB597, 0.3 mg/kg	i.p.	Compare impact of THC and URB597 on mPFC and vHIP neuronal activity in PCP-treated rats and saline-treated controls	Reduced baseline firing rates in PCP-treated rats compared to controls; THC reduced mPFC alpha power in only controls, enhanced mPFC and HIP delta power in all rats, and enhanced mPFC firing rates in PCP-treated rats; For all rats, URB597 enhanced mPFC gamma power and reduced HIP delta power and mPFC-HIP delta coherence
Atallah and Scanziani (13)	–	Wistar rats	–	–	Determine cellular mechanisms involved in phase-shifts of HIP oscillations	HIP (CA3) gamma amplitudes predict the interval to next cycle; synaptic inhibition is proportional to synaptic excitation during each cycle
Barz et al. (14)	Schizophrenia	NRG1 +/– KO mice	–	–	Examine sensory-related spiking and gamma oscillations in somatosensory cortex of NRG1 mutant mice and WT controls using whisker stimulation	Elevated baseline firing and reduced gamma power in NRG1 mouse barrel cortex, compared to controls
Dzirasa et al. (15)	Bipolar disorder	Clock-Δ19 mice	–	–	Record from NAc, PrlC, and VTA in ClockΔ19 mice and littermate controls while they explore a novel environment	ClockΔ19 mice exhibit baseline NAc low-gamma phase coupling and neuronal entrainment deficits; lithium partially ameliorated deficits
Gazit et al. (16)	Major depressive disorder	Flinders Sensitive Line (FSL) Sprague Dawley rats	–	–	Examine impact of DBS on VTA coherence and depressive-like behaviors	FSL rats exhibit reduced baseline gamma coherence; DBS restored coherence and rescued behavioral deficits
Goodwill et al. (17)	Major depressive disorder	Early-life stress via limited bedding stress in Long Evans rats	–	–	Assess sex differences in depressive-like behaviors	Females exhibited depressive-like behaviors in adulthood after early-life stress, while males did not
Hajos et al. (18)	–	Sprague Dawley rats	CP-55940, 0.1 mg/kg; AM-251, 3 mg/kg	i.v.	Measure cannabinoid-induced disruptions in auditory sensory gating and neurophysiological correlates in the EC and HIP; determine whether they are CB1R-mediated	CP-55940 reduced EC theta power, EC and HIP gamma power, and EC and HIP theta coherence; AM251 reversed EC gamma power and EC, HIP theta coherence deficits
Hudson et al. (19)	–	Sprague Dawley rats	THC, 10 and 100 ng; CBD, 10 and 100 ng	infusion	Investigate impact of THC and CBD in vHIP on VTA neural circuit activity and emotional memory	THC enhanced VTA delta, beta, and gamma power, and reduced VTA firing rates; CBD ameliorated effects on VTA firing rates and delta power
Iniguez et al. (20)	Major depressive disorder	Chronic social defeat stress in c57BL/6 mice	–	–	Determine whether adolescent CSDS produces a depressive-like phenotype	Adolescent CSDS produces depressive-like behaviors in c57BL/6 mice
Iturra-Mena et al. (21)	Major depressive disorder	Chronic social defeat stress in Sprague Dawley rats	–	–	Determine the impact of CSDS on NAc oscillations during social interactions	NAc gamma power was enhanced in control rats, but not CSDS rats, during social interaction
Khalid et al. (22)	Major depressive disorder	Chronic restraint stress in c57BL/6 mice	–	–	Investigate functional connectivity in cortical regions	Increased cortical gamma coherence after chronic stress exposure, dissipated with remission of depressive-like behavior
Lecca et al. (23)	Schizophrenia	Maternal immune activation in Sprague Dawley rats	THC, 2.5 mg/kg (PND 45–47); 5 mg/kg (PND 48–51); 10 mg/kg (PND 52–55)	i.p.	Examine the impact of adolescent cannabinoid exposure on neurophysiological deficits in adult rats	Reduced number of active VTA DA neurons in poly I:C rats compared to controls and reduced mPFC serotonergic burst activity in controls after WIN-55,212-2
Lee et al. (24)	Schizophrenia	Neonatal ventral hippocampal lesion in Long Evans rats	–	–	Measure dysfunctional neural synchrony in NVHL rats and restore deficits by normalizing synchrony with ethosuximide	NVHL rats exhibit increased amplitudes and spiking activity, reduced theta and beta coherence during place avoidance behavior; ethosuximide reduced spiking activity and attenuated coherence deficits
Linge et al. (25)	–	Olfactory bulbectomy in c57BL/6 mice	CBD, 50 mg/kg; AM251, 03 mg/kg	i.p.	Examine acute and chronic effects of CBD on depressive-like behavior and PFC serotonin/glutamate activity	Acute and chronic CBD reversed behavioral hyperactivity and increased PFC serotonin and glutamate levels in model mice
Moussa-Tooks et al. (26)	Major depressive disorder	Early-life stress via limited bedding stress in Long Evans rats	–	–	Demonstrate early-life stress sex-dependently down-regulates cerebellar endocannabinoids in adulthood and impacts behavior	Early-life stress produced sex-specific changes in endocannabinoid expression and impaired behavior on OR and social recognition
Nelong et al. (27)	–	Sprague Dawley rats	THC, 10 mg/kg	Vapor	Measure the acute effects of THC vapor exposure on LFPs in the dStr, OFC and PFC of rats using a within subject design	THC vapor exposure suppressed oscillatory power and coherence, most notably in the gamma band; this effect was detected after the 7 day washout period
Nguyen et al. (28)	–	Sprague Dawley rats	THC; 25, 50, 100, 200 mg/mL	Vapor	Validate THC vapor administration protocol in rats using measures from the cannabis tetrad	THC vapor exposure predictably reduced body temperature, locomotor activity, and nociception in male and female rats
Park et al. (29)	Major depressive disorder	Restraint plus tail shock (RTS) in Sprague Dawley rats	WIN55,212-2, 1 mM; AM251, 5 μM	Bath	Detect and measure stress-induced changes of LTD in the LHb	Low- and moderate-frequency stimulation induced LTD in the LHb; acute stress exposure prevented only Low-frequency-induced LTD in the LHb
Raver and Keller (30)	-	CD-1 mice	THC, 5 mg/kg; WIN55,212-2, 1 or 2 mg/kg; AM251, 0.3, 0.5, 1, or 2 mg/kg	i.p.	Assess the impact of adolescent WIN55-212,2 and THC exposure on cortical oscillations and memory in adult rats	Chronic adolescent (but not adult) WIN55-212,2 exposure attenuates adult cortical oscillations
Renard et al. (31)	–	Sprague Dawley rats	CBD, 100 ng	Infusion	Assess antipsychotic-like actions of CBD on amphetamine-induced VTA DA dysfunction	CBD attenuated the amphetamine-induced increase in VTA DA firing rates
Renard et al. (32)	–	Sprague Dawley rats (adolescent)	THC, 2.5 mg/kg; Days 1–3; 5 mg/kg; Days 4–7; 10 mg/kg, Days 8–11	i.p. (twice daily)	Examine the impact of adolescent THC on PFC GABA and VTA DA activity in adult rats	Adolescent THC attenuated PFC GABA activity, increased neuronal firing, disrupted gamma oscillations, and increased VTA DA activity
Sauer et al. (33)	Major depressive disorder	DISC1 mice	–	–	Characterize PrlC network dysfunction in DISC1 mice	DISC1 mice exhibit reduced PrlC theta and low gamma power
Seewoo et al. (34)	Major depressive disorder	Chronic restraint stress in Sprague Dawley rats	–	–	Assess functional connectivity changes related to depressive-like behaviors using MRI	MRI showed hypoconnectivity in salience and interoceptive networks, and hyperconnectivity between the cingulate cortex and multiple corticolimbic regions
Segev et al. (35)	Major depressive disorder	Chronic mild variable stress in Sprague Dawley rats	WIN55,212-2, 0.5 mg/kg (i.p.); 5 mg/side (infusion); AM-251, 0.3 mg/kg	i.p., infusion	Assess whether WIN55-212,2 ameliorates CMS-induced changes in HIP-NAc LTP, and whether this is CB1R-dependent	WIN55-212,2 treatment prevented CMS-induced deficits in HIP-NAc LTP, an effect lost with AM251 administration
Seillier et al. (36)	Schizophrenia	Subchronic PCP-treated Sprague Dawley rats	THC, 0.1, 0.3 or 1 mg/kg; AM251, 1 mg/kg	i.p.	Determine the effects of THC on social withdrawal in PCP-treated rats and neurophysiological correlates	In controls, THC dose-dependently produced social interaction deficits and aberrant VTA DA neuronal activity; in PCP-treated rats, only the lowest dose of THC reversed PCP-induced deficits
Sigurdsson et al. (37)	Schizophrenia	Df(16) A+/– mice	–	–	Examine functional connectivity between the HIP and the PFC in Df(16) A+/– mice during a memory task	WT mice exhibit increased HIP-PFC coherence during working memory; Df(16) A+/– mice exhibit reduced coherence
Taffe et al. (38)	-	Sprague Dawley rats; Wistar rats	THC, 5, 10, 20 or 30 mg/kg	i.p.	Compare the effects of THC vapor exposure between Wistar and Sprague Dawley rats using measures from the cannabis tetrad	Hypothermia was more pronounced in Sprague Dawley rats compared to Wistar rats, while antinociception did not differ between strains
Tchenio et al. (39)	Major depressive disorder	Maternal separation in c57BL/6 mice	–	–	Determine whether restoring LHb function ameliorates a depressive-like phenotype	LHb neuronal hyperexcitability is ameliorated by chemogenetic modulation and DBS
Valvassori et al. (40)	Bipolar disorder	Wistar rats	CBD, 15, 30 or 60 mg/kg	i.p.	investigate the effects of CBD on an amphetamine-induced, manic-like phenotype	CBD reversed amphetamine-induced damage in the HIP and enhanced BDNF expression. CBD administered before amphetamine prevented damage
Voget et al. (41)	Major depressive disorder	Flinders Sensitive Line (FSL) rats	–	–	Characterize neural circuit activity changes in the PFC, NAc, Cg, and STN in FSL rats	Compared to controls, FSL rats exhibit reduced mPFC and NAc alpha, beta, and gamma power, reduced STN alpha and beta power, and enhanced STN gamma power
Young et al. (42)	Schizophrenia	Subchronic PCP-treated Wistar rats	–	–	Assess changes in mPFC neural circuit oscillations in PCP-treated rats	PCP-treated rats exhibit reduced PFC theta power and enhanced PFC coherence

As described below, cannabinoids produce distinct changes in neural circuit electrophysiological activity that are similar to those observed in patients with serious mental illnesses, as well as rodent models of these illnesses (43, 44). Neural circuit oscillatory activity arises from the summed electrical activity of networked neurons and is apparent in electrophysiological recordings from human subjects as well as non-human research animals, with various frequencies corresponding to certain functions (45–49). These frequencies and their associated functions are simplified as follows: delta oscillations (0.5–4 Hz) are associated with signal detection and decision making; theta oscillations (4–7 Hz) are associated with episodic memory and memory retrieval; alpha oscillations (8–12 Hz) are associated with semantic memory and attention; beta oscillations (13–29 Hz) are associated with motor control as well as attention, and sensory filtering; gamma oscillations (30–90 Hz) are associated with attention, sensation, perception, memory, and conscious awareness (45–49). Aberrant oscillatory patterns thus correlate with different brain states, including those resulting from cannabinoid exposure or contributing to a serious mental illness-related symptom; dysfunctional patterns often reflect deficits in behavior and cognition. While we will ultimately identify similarities between cannabinoid-induced oscillatory changes and pathological changes associated with serious mental illnesses, cannabis exposure alone can produce phenotypes that overlap with some psychopathology, making it important to disentangle the effects of cannabis alone from its interactions with the psychopathology-associated circuit dysfunctions.

## Modeling Cannabis Use and Serious Mental Illness

### Cannabis Use

Cannabis users inhale smoke or vapor from crudely burning cannabis flower or by vapourizing it at greater temperatures (50). Researchers modeling cannabis use employ various administration routes (i.e., injection, vaporization, oral ingestion, or inhalation) of cannabis plant components and synthetic cannabinoids, including cannabinoid-type 1 receptor (CB1R) agonists such as CP-55940 and WIN55,212-2. CB1R antagonists rimonabant and AM251 are also used to assess the involvement of the endocannabinoid system (eCB) in drug effects and psychopathology (51). Examining cannabinoids beyond their action at CB1R sites, however, is imperative, as interactions also occur via CB2R-dependent mechanisms (52–55), as well as non-cannabinoid receptor mediated mechanisms (56–58). Moreover, the use of CB1 receptor agonists to model the exposure to cannabis-derived cannabinoids may also have limited utility due to their limited pharmacological profile. Indeed, the effects of cannabis arise from combined constituent activity (59), not the action of a single ligand-receptor interaction, and thus future preclinical studies, unless purely pharmacological, must examine the combined effects. Also, since injections do not capture human use patterns, a recent concerted effort to establish more translationally-relevant delivery methods (e.g., vaping) for Δ9-tetrahydrocannabinol (THC) and cannabidiol (CBD), as well as the many other constituents in the cannabis plant, has begun (28, 38, 60).

### Schizophrenia

Schizophrenia is a complex neuropsychiatric illness characterized by severe dysfunctions including delusions, hallucinations (and other “positive” symptoms), social withdrawal (and other “negative” symptoms), and deficits in memory and sensory processing (and other “cognitive” symptoms) (61). Considering the phenotypic complexity of schizophrenia, a rodent model that singularly recapitulates the human condition does not exist. Rather, various models (i.e., genetic, neurodevelopmental, and pharmacological) produce dysfunctions that capture some parts of the disease symptoms. These models usually demonstrate positive-like (e.g., amphetamine-induced hyperlocomotion, deficits in prepulse inhibition mediated via enhanced dopamine signaling), negative-like (e.g., social withdrawal), and cognitive-like behavioral dysfunctions (e.g., deficits in attention and working memory) (62, 63). As disrupted-in-schizophrenia 1 (DISC1) gene was one of the first genes implicated in schizophrenia, many transgenic models targeting this gene exist (64, 65). Similarly, knocking out the NRG1 gene, which is implicated in schizophrenia, is also used to study schizophrenia-like behaviors and circuit dysfunctions (66). Neurodevelopmental models are created by altering rodent neurodevelopment, by either administering polyriboinosinicpolyribocytidilic acid (poly I:C) to pregnant dam to produce maternal immune activation (23, 67), or via bilateral lesioning of neonatal ventral hippocampi (NVHL) using ibotenic acid (63, 68, 69). Pharmacological models involve administering a compound to modify neurotransmission; for example, phencyclidine (PCP) produces psychotomimetic effects akin to the symptoms of schizophrenia (42, 70).

### Major Depressive Disorder

Individuals diagnosed with major depressive disorder present with symptoms such as persistent negative affect, anhedonia, as well as disturbed sleep and appetite (71). In rodents, depressive-like symptoms are produced using genetic models or by stress exposure through chronic mild/variable stress, social defeat stress, or early life stress. Genetic models include the Flinders Sensitive Line (FSL) and Wistar Kyoto rats, which exhibit phenotypical similarities to major depressive disorder in humans (72, 73). Wistar Kyoto rats are used specifically for modeling treatment-resistant depression (74, 75). Chronic mild/variable stress involves daily exposure to various stressors (e.g., tail suspension, restraint, electrical shock) (20). Social defeat stress involves repeatedly exposing a submissive rodent to a dominant conspecific (76), while early life stress involves separating neonates from dams or altering the rearing environments (17, 77). These modifications produce depressive-like behaviors in rodents, including reduced exploration, reduced sucrose preference (reflecting an anhedonic state), and reduced escape attempts (reflecting amotivation or despair) (78). Interestingly, CB1R-deficient mice are used to model major depressive disorder (79–81). Social defeat stress in mice also reduces CB1R expression in the basolateral amygdala, a brain region involved in the pathophysiology of major depressive disorder, while knocking down or knocking out CB1R expression in mice enhances stress susceptibility (81, 82).

### Bipolar Disorder

The symptoms of bipolar disorder are characterized by cyclic changes in mood, motivation, and attention, ranging from periods of manic to depressed symptoms (83). Although producing a model comprising the complete range of symptoms has proven difficult, rodent models of psychosis, depression, and diurnal disruption are often used to model aspects of bipolar disorder (84, 85). The ClockΔ19 transgenic mouse model shows promise as a heuristic model of bipolar disorder, having both face (behavioral cycling, hyperactivity) and predictive validity, as lithium administration decreases bipolar-like behaviors in this model (85). The dopamine transporter knock-down (DAT-KD) mouse is also used to model mania-like behaviors (enhanced motivation, hyperactivity) associated with human bipolar disorder (86–90). DAT knock-out (DAT-KO) mice are also sometimes used, but present with growth defects and hypoplasia. The DAT-KD mouse was subsequently developed to avoid these undesirable attributes (86, 91, 92).

## Rodent Models of Serious Mental Illness Exhibit Aberrant Neural Circuit Activity

### Schizophrenia

**Figure 1:**
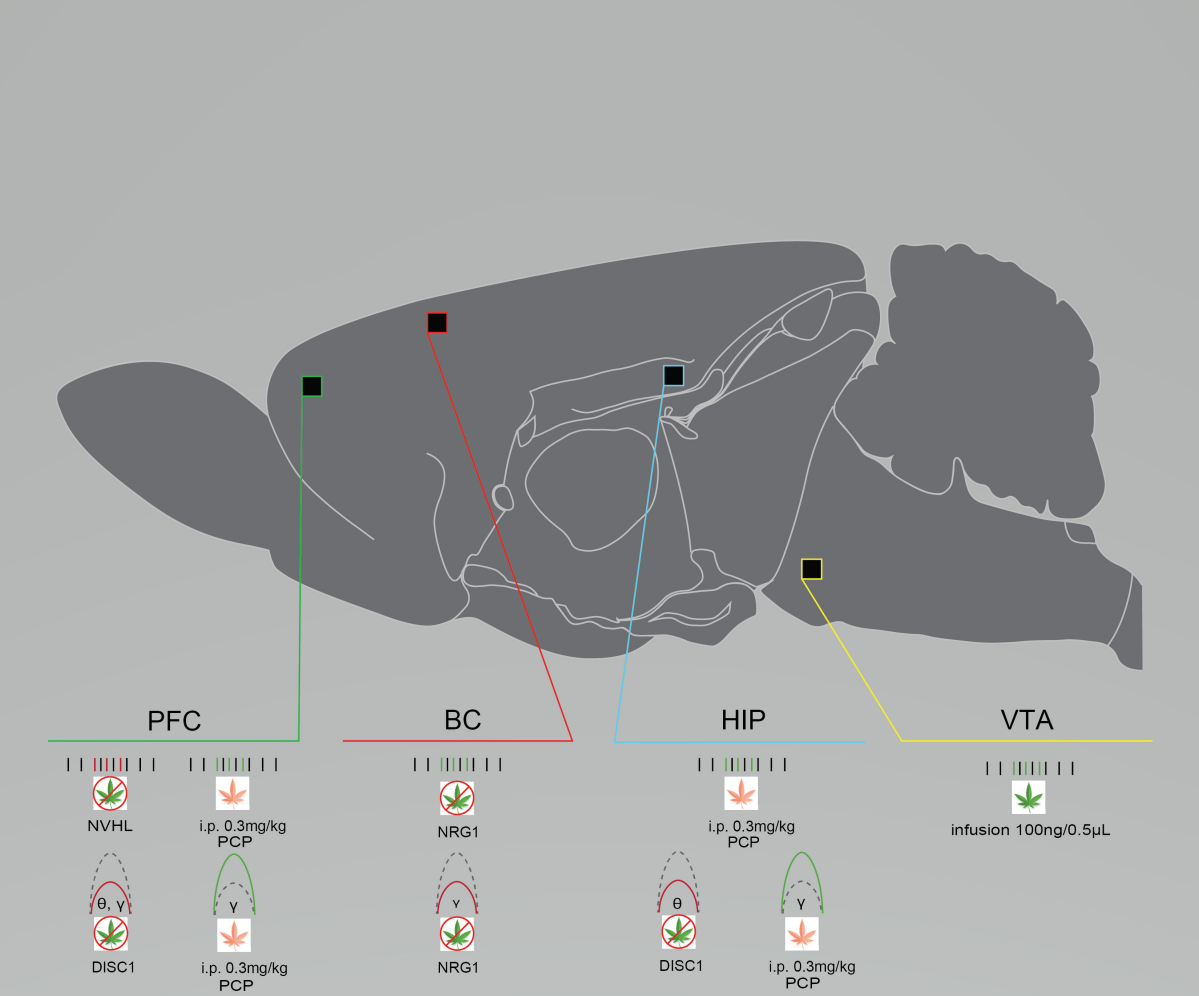
Rodent models of schizophrenia exhibit neural circuit disruptions and eCB modulation enhances this activity. Graphical summary of preclinical investigations demonstrating neural circuit disruptions induced by cannabinoid exposure in rodents used to model schizophrenia. Rodents modeling schizophrenia exhibit reduced baseline PFC neuronal firing rates, reduced baseline PFC, BC, and HIP spectral power, and enhanced baseline BC neuronal firing rates. THC exposure increases neuronal firing rates in the VTA after infusion. URB597 exposure increases neuronal PFC and HIP firing rates and spectral power. BC, barrel cortex; DISC1, DISC1 KO genetic mouse model of schizophrenia; HIP, hippocampus; NRG1, NRG1 knock-down genetic mouse model of schizophrenia; NVHL, NVHL rat model of schizophrenia; PCP, Phencyclidine rat model of schizophrenia; PFC, prefrontal cortex/prelimbic cortex; VTA, ventral tegmental area. Green: Increase; Red: Decrease.

Patients with schizophrenia exhibit reduced resting-state and evoked theta and gamma power, as well as decreases in beta and gamma coherence spanning various brain regions (93–96). Similar alterations are also evident in preclinical models (Figure 1). Single-unit and local field potential (LFP) recordings from layers II/III and IV in the barrel cortex of anesthetized NRG1 knock-out mice and wild-type (WT) controls showed that NRG1 mice exhibit reduced gamma power. This reduction was also associated with reduced gamma signal-to-noise ratio and phase-locking (for all frequencies below 50 Hz), underpinned by enhanced firing rates, possibly demonstrating mechanistic dysfunction that occurs in patients with schizophrenia (14). In DISC1 mice, *in vivo* LFPs from the prelimbic cortex (PrlC) and hippocampus (HIP) showed reduced theta power in the HIP and PrlC and low gamma (30–50 Hz) power only in the PrlC (33) (Figure 1). PrlC-HIP coherence remained intact as described previously (37). NVHL rats showed reduced theta and beta coherence in the dorsal HIP (dHIP) while medial prefrontal cortex (mPFC) coherence remained intact. Baseline dysfunctions in inter-spike timing, wave duration, spike to valley voltage, and wave energy were also apparent, again possibly revealing causal mechanisms of oscillatory dysfunction in human schizophrenia (24) (Figure 1). Notably, gamma power suppression is consistently demonstrated across different models, and in human subjects.

### Major Depressive Disorder

**Figure 2:**
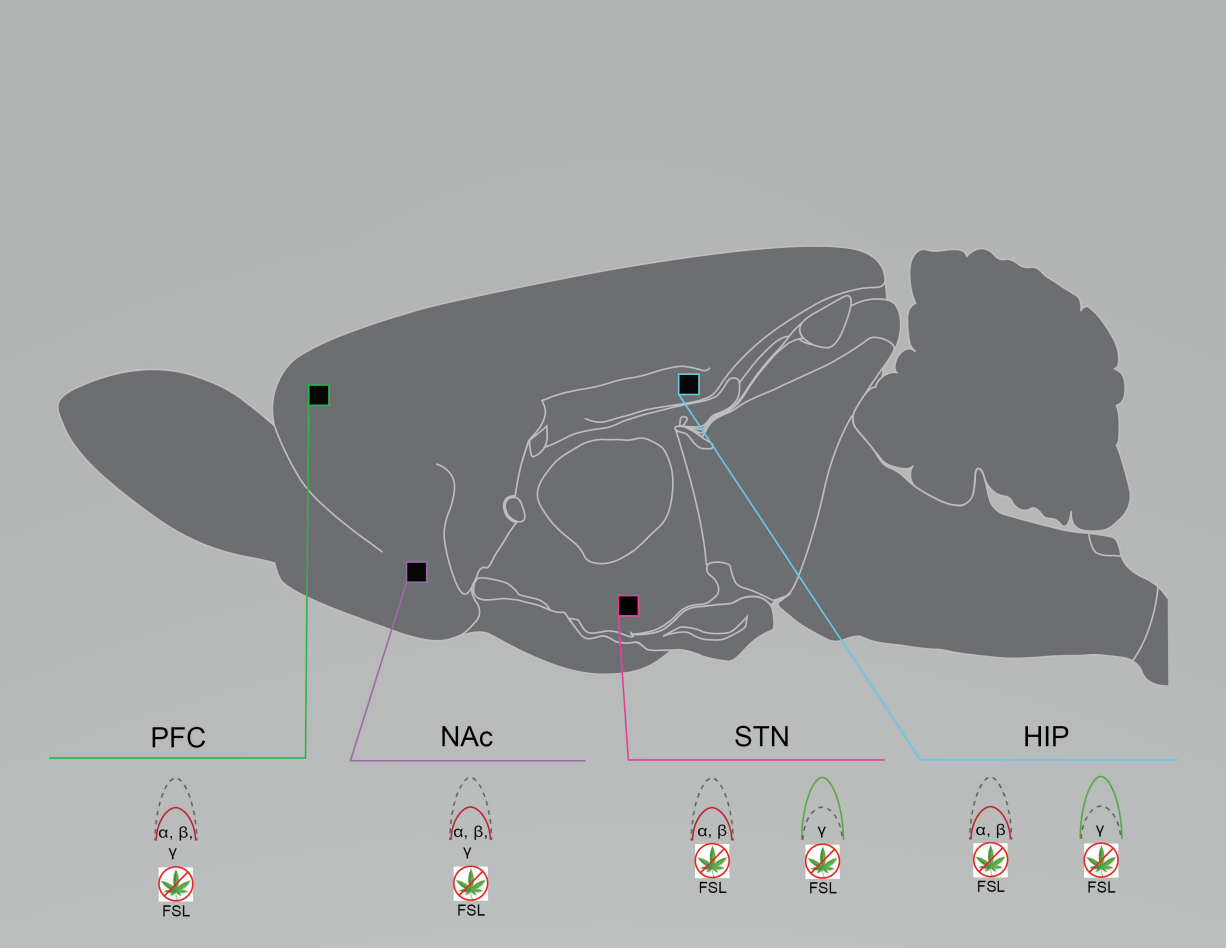
Rodent models of major depressive disorder exhibit region- and frequency-specific neural circuit disruptions. Graphical summary of preclinical investigations demonstrating neural circuit disruptions induced by cannabinoid exposure in rodents used to model major depressive disorder. Rodents modeling major depressive disorder exhibit reduced baseline PFC, NAc, and STN spectral power in the lower frequency bands and enhanced baseline STN and HIP spectral power in the higher frequency bands. FSL, Flinders Sensitive Line rat model of major depressive disorder; HIP, hippocampus; NAc, nucleus accumbens; PFC, prefrontal cortex/prelimbic cortex; STN, subthalamic nucleus. Green: Increase; Red: Decrease.

Reductions in alpha, theta, and gamma oscillations appear in patients with major depressive disorder, and in genetic and stress-induced preclinical models (97, 98). In anesthetized FSL rats, LFPs from the mPFC, nucleus accumbens (NAc) shell, and the subthalamic nucleus (STN) all exhibited reduced alpha and beta power compared to controls, while theta and high gamma power remained intact; low gamma power in FSL rats differed by region, with reduced power in the mPFC and NAc and enhanced power in the STN (41) (Figure 2). Although alpha, theta, and gamma suppression is apparent in patients with major depressive disorder, preclinical models demonstrate that this suppression is more varied and region-specific. Similarly, rats exposed to chronic social defeat stress (CSDS) also exhibit aberrant gamma activity. LFP recordings from the NAc of CSDS rats and unstressed controls were acquired during social interaction or free exploration. High gamma power was enhanced in controls during social interaction, whereas CSDS rats did not exhibit any change in gamma power during either activity (21). In the chronic restraint stress (CRS) mouse model, LFPs from the frontal, somatosensory, parietal, and visual cortices were captured at 7 and 21 days post-stress exposure. At the 7 day time-point, CRS mice exhibited only enhanced coherence across all frequencies compared to baseline. At the 21 day time-point, the enhanced delta and gamma coherence disappeared. Thus, gamma was restored with remission of a depressive-like phenotype (22), which indicates that aberrant gamma power or coherence may be a viable biomarker for major depressive disorder. As is the case with schizophrenia, dysfunctional gamma is common across various rodent models and in patients with major depressive disorder.

### Bipolar Disorder

**Figure 3:**
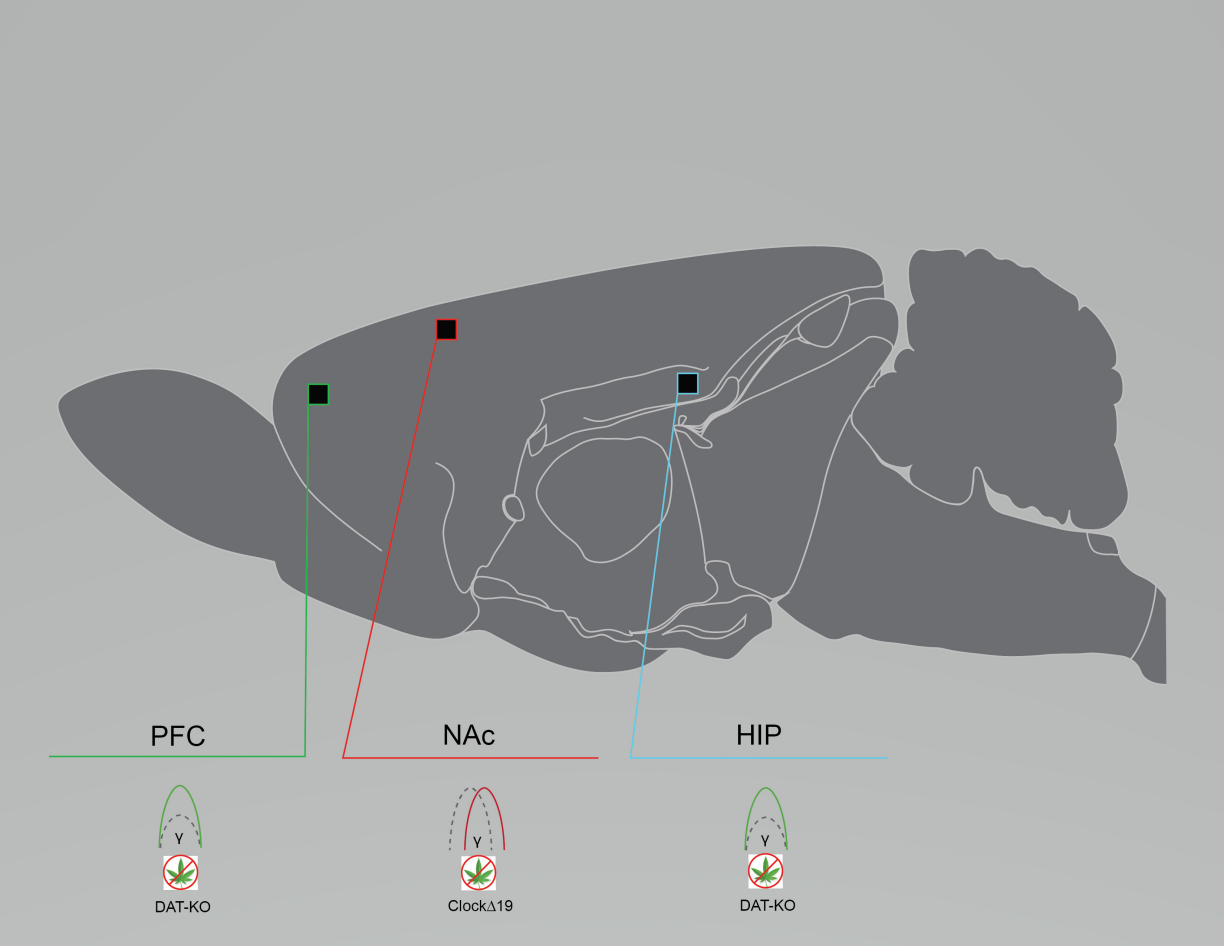
Rodent models of bipolar disorder exhibit reduced baseline neural circuit spectral power and coherence. Graphical summary of preclinical investigations demonstrating neural circuit disruptions induced by cannabinoid exposure in rodents used to model bipolar disorder. Rodents modeling bipolar disorder exhibit reduced baseline PFC and HIP spectral power, and reduced baseline NAc coherence. CLOCKΔ19, CLOCKΔ19 genetic mouse model of bipolar disorder; DAT-KO, DAT-KO genetic mouse model of bipolar disorder; HIP, hippocampus; NAc, nucleus accumbens; PFC, prefrontal cortex/prelimbic cortex. Green: Increase; Red: Decrease.

Patients with bipolar disorder exhibit various aberrations in oscillatory activity, including enhanced or reduced alpha power, enhanced beta power, enhanced alpha, beta, and gamma coherence, and reduced evoked frequencies (30, 99–103). Rodent models also exhibit altered oscillatory activity, although the literature is sparse. LFPs from the NAc, the PrlC, and the ventral tegmental area (VTA) in freely-exploring ClockΔ19 mice and littermate controls demonstrated that ClockΔ19 mice exhibited reduced low gamma to delta phase-coupling, as well as intact high gamma to delta phase-coupling, in the NAc. Low and high gamma coupling also appeared to be reduced in the PrlC and the VTA of ClockΔ19 mice. ClockΔ19 mice also exhibited disrupted phase-locking of NAc neuron firing to delta oscillations. Contradicting what is observed in the clinical literature, a difference in power between ClockΔ19 and controls was not observed (Figure 3). In support of altered oscillatory activity being relevant for human bipolar disorder is preclinical evidence that lithium chlorate (600 mg/L of water) administration, the primary prescribed treatment for patients with bipolar disorder, increased low gamma phase-coupling and ameliorated phase-locking deficits in the NAc of ClockΔ19 mice (15). In DAT-KD mice, whole-cell patch clamp recordings of dorsal striatum (dStr) glutamatergic neurons revealed shorter half-amplitude durations and faster decay times (86). This may be contributing to the altered phase coupling reported by Dzirasa et al. (92) in DAT-KO mice. LFPs from the dHIP and the mPFC of DAT-KO mice also demonstrated enhanced dHIP-PFC gamma coherence compared to controls (92) (Figure 3). Although the preclinical literature is sparse and does not completely reflect what is observed in clinical studies of patients with bipolar disorder, reduced oscillatory activity in the gamma frequency range is again a shared feature across models and human subjects.

## Cannabinoid Exposure in Rodents Alters Neural Circuit Activity

**Figure 4:**
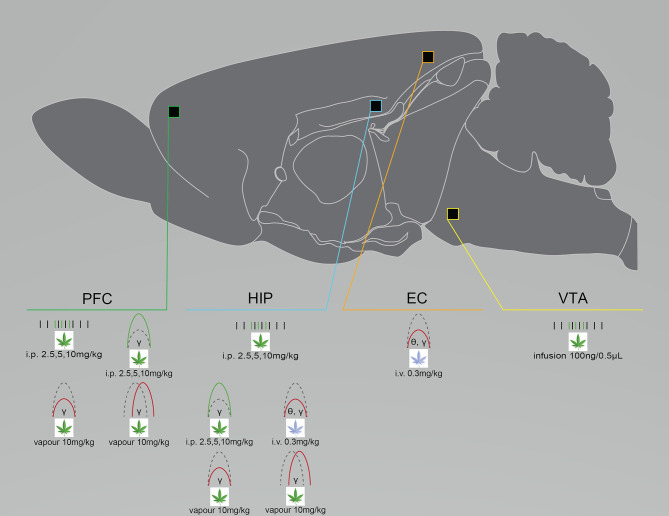
Cannabinoid exposure disrupts neural circuit activity differently depending on administration route and dose, as well as the chosen cannabinoid. Graphical summary of preclinical investigations demonstrating neural circuit disruptions induced by cannabinoid exposure in healthy rodents. THC exposure increases neuronal firing rates in the PFC, HIP and VTA after i.p. injection or infusion, enhances spectral power after i.p. injection and suppresses spectral power after i.v. injection or vapor exposure, and suppresses PFC and HIP coherence after vapor exposure. WIN-55 exposure suppresses HIP and EC coherence after i.v. exposure. Studies showing no effect of cannabinoid exposure were not included. CP-55, CP-55940; EC, entorhinal cortex; HIP, hippocampus; PFC, prefrontal cortex/prelimbic cortex; VTA, ventral tegmental area. Green: Increase; Red: Decrease.

Cannabinoid-induced neural circuit dysfunctions in animal studies and otherwise healthy humans are similar to those in patients with a serious mental illness (18, 44, 104, 105). THC exposure acutely suppresses gamma power and increases cortical noise in otherwise healthy individuals; changes that are associated with increased symptoms of psychosis (43, 106, 107). Acute THC exposure also alters oscillatory activity in patients with schizophrenia, with evidence showing it suppresses and enhances resting-state theta and low gamma power, respectively (108), while chronic use in patients suppresses amplitudes of auditory evoked potentials (109, 110). Renard et al. (32) injected adolescent, male Sprague Dawley rats with escalating doses of THC (2.5–10 mg/kg, i.p.) and recorded glutamatergic and dopaminergic (DA) neurons in the PFC and the VTA from anesthetized adult rats. Compared to vehicle-treated controls, THC increased PFC glutamatergic firing and burst rates, and high gamma (61–80 Hz) power during desynchronized states. It also increased VTA DA firing frequencies and spontaneous bursting (32) (Figure 4). In adult anesthetized rats, Skosnik et al. (44) intravenously administered vehicle, CP-55940 (0.3 mg/kg), or CP-55940 + AM251 (3 mg/kg), while LFP and auditory evoked potentials (AEPs) were recorded from the HIP and entorhinal cortex. CP-55940 reduced AEP theta and gamma power, which was partially reversed by AM251 co-administration, suggesting THC-induced changes are partly CB1R mediated (44) (Figure 4). We recently demonstrated that rats acutely exposed to THC vapor have oscillatory changes lasting longer than 1 week in the PFC, the orbitofrontal cortex (OFC), and the dStr after either vehicle or THC vapor (10 mg/kg) administration. Reduced gamma power was measured in all brain regions of THC-treated rats, compared to controls. Reduced dStr-OFC and OFC-PFC gamma coherence was also observed, and within-subject comparisons of rats exposed to THC in week 1 and vehicle in week 3 demonstrated persisting gamma suppression (27) (Figure 4). Infusions of THC (100 ng/0.5 μL) and CBD (100 ng/0.5 μL), either alone or in combination, directly into the vHIP of anesthetized male Sprague Dawley rats while recording VTA DA and non-DA GABAergic neurons demonstrated that THC enhanced VTA DA firing and bursting rates, compared to controls (Figure 4). Both THC and CBD enhanced firing frequencies of VTA non-DA neurons compared to controls, while only THC administration enhanced beta, gamma, and low delta power; THC also increased behavioral measures of fear responsivity, morphine conditioned place preference, and sucrose preference. CBD reversed all THC-induced changes (except for sucrose preference, which it enhanced) when co-administered (19), which supports evidence of CBD as a potential antipsychotic, antidepressant and anxiolytic (25, 58, 111).

## Differential Effects of Cannabinoid Exposure in Rodent Models of Serious Mental Illness

### Schizophrenia

Cannabinoid exposure differentially affects neural circuit activity in rodent models of schizophrenia, in a dose- and region-dependent manner, and when comparing model animals to controls. Seillier et al. (36) examined whether THC exposure dose-dependently alters baseline VTA DA activity in PCP-treated or control rats. In PCP-treated rats, a reduced number of active VTA DA neurons at baseline was evident when compared to controls [a result that contradicts existing evidence of augmented DA population activity (11)]. The low dose (0.1 mg/kg, i.p.) of THC enhanced VTA DA population activity in PCP-treated rats, while the high dose (1 mg/kg, i.p.) of THC had no effect. In control rats, the low dose of THC reduced the number of active VTA DA neurons to baseline levels observed in PCP-treated rats. Extending this result to examine the involvement of the eCB in additional brain regions, Aguilar et al. (12) used the subchronic PCP-treated rat model to examine changes in neural circuit activity after exposure to THC (1 mg/kg, i.p.) or URB597 (0.3 mg/kg, i.p.), a fatty-acid amide hydrolase inhibitor, administered to awake, behaving rats. Single-unit and LFP recordings from the PFC and vHIP were taken prior to injection and at 30 min intervals post-administration. THC reduced mPFC firing rates in controls, without producing any effect in rats treated with PCP. URB597 increased mPFC firing rates in rats treated with PCP, compared to controls (Figure 4). THC also did not produce any effect on firing rates in the vHIP. PFC and vHIP baseline oscillatory activity was similar between PCP-treated rats and controls. THC administration also did not alter oscillatory activity in either group, which the authors attribute to the low dose of THC used. URB597 administration increased gamma power in the mPFC and decreased delta power in the vHIP for both groups (Figure 4). THC and URB597 produced opposing effects on mPFC-vHIP coherence, increasing, and decreasing delta coherence, respectively, for both groups; thus, also revealing that eCB modulation is differentially impacted by various modulations (12). Cannabinoid-induced alterations to neural activity may also be age-dependent; Lecca et al. (23) used the poly I:C rat model to examine whether adolescent cannabis exposure alters adult VTA DA neuron sensitivity. Adolescent poly I:C rats and controls were administered either vehicle or escalating doses of THC (2.5–10 mg/kg, i.p.) for 11 days. Once in adulthood, recordings were captured from the VTA of anesthetized rats. In Poly I:C rats, the number of spikes per burst and the intra-burst frequency of VTA DA neurons were reduced, compared to controls, which was ameliorated by adolescent THC exposure. Taken together, THC exposure may enhance DA population activity in rodent models of schizophrenia only at higher doses and in particular brain regions, whereas THC suppresses DA population activity in control animals. This possibly reflects an underlying mechanism supporting data from patients with schizophrenia that demonstrates THC acutely enhances oscillatory power in patients and suppresses power in controls (43, 108).

### Major Depressive Disorder

As was observed in the rodent models of schizophrenia described above, cannabinoid exposure enhances neural circuit activity in rodent models of major depressive disorder, and this effect seems more pronounced compared to control animals. Abush and Akirav (10) examined the longevity of cannabinoid-induced neural changes in a rat model of major depressive disorder. Male, Sprague-Dawley rats underwent daily chronic restraint stress (CRS) and administration of vehicle or WIN55,212-2 (1.2 mg/kg, i.p.). Thirty days after stress exposure, LTP recordings were captured from the NAc shell of anesthetized rats. Stress-exposed rats exhibited reduced LTP in the NAc while stress-exposed rats administered WIN55,212-2 were like unstressed controls, suggesting WIN55,212-2 rescued stress-induced LTP deficits. Within-subject comparisons of the unstressed, WIN55,212-2-treated rats revealed that WIN55,212-2 enhanced LTP compared to baseline. WIN55,212-2 + AM251 co-administration ameliorated WIN55,212-2 enhancements of LTP (10). These results were also reproduced using the chronic mild/variable stress model of major depressive disorder, as WIN55,212-2 administration (0.5 mg/kg, i.p.) prevented stress-induced reductions in LTP in the NAc and this was reduced by AM251 (0.3 mg/kg, i.p.) co-administration (35). Unlike the rodent models of schizophrenia mentioned above, WIN55,212-2 exposure produced a similar effect in control rats, albeit to a lesser extent than CRS rats (10). Another study, using restraint and tail-shock stress in Sprague Dawley rats to induce a depressive-like phenotype, demonstrated using slice electrophysiology that WIN55,212-2 (1 μM bath) induces long-term depression (LTD) in the lateral habenula of both stress-exposed rats and controls, a brain region implicated in the pathogenesis of major depressive disorder (29). Taken together, these results indicate that WIN55,212-2 exposure alters LTP and LTD mechanisms in both model and control rats.

### Bipolar Disorder

Studies using validated models of bipolar disorder to examine cannabinoid-induced functional changes in neural circuit electrophysiological activity are very limited, so we have included an alternate measure of neural circuit alteration below. One study investigated the impact of CBD on mania-like behavior and neurobiology using amphetamine-induced hyperlocomotion in male Wistar Kyoto rats, to model mania in bipolar disorder. Rats were treated with escalating doses of CBD (15–60 mg/kg, i.p.) before, or after, exposure to D-amphetamine (2 mg/kg) to produce an acute manic episode. CBD did not affect amphetamine-induced hyperlocomotion in this rat model, which contradicts existing literature and may reflect innate differences in Wistar Kyoto rats (31). The moderate dose of CBD (30 mg/kg) increased HIP BDNF expression and rescued amphetamine-induced damage (possibly due to its effects as an anti-oxidant) when given after amphetamine exposure, whereas CBD pre-treatment had no effect on BDNF expression in the HIP, showing a protective effect of CBD on the biochemical changes associated with this model of bipolar disorder, only when administered post-treatment (40). This exemplifies how the effect of cannabinoid exposure depends on the chosen administration protocol. Moreover, given the involvement of BDNF signaling in coordinating gamma activity (112), we expect that future studies examining oscillatory activity in this rodent model of bipolar disorder may reveal reductions in gamma activity induced by cannabinoid exposure.

## Discussion

Understanding the pathophysiology of co-occurring cannabis use and serious mental illnesses remains a challenge in neuropsychiatry. Aberrant theta, alpha, beta, and gamma oscillations are observed after cannabinoid exposure in humans and in mental illness, while cannabinoid exposure differentially impacts the symptoms of the afore-mentioned mental illnesses. Similarly, preclinical models exhibit suppressed baseline neural circuit oscillatory activity, while cannabinoid exposure produces differential effects on electrophysiological neural circuit activity in otherwise untreated animals and has mixed effects in preclinical models on oscillatory activity, neuroplasticity, and neuronal firing rates. That said, our mini-review (albeit not exhaustive) shows that aberrant gamma activity is consistently observed. Whether aberrant gamma is etiological or only a consequence remains contested; however, the rescuing effect of gamma modulation implies that it is involved in mediating pathophysiological mechanisms (16, 113).

Gamma oscillations arise from competing excitatory and inhibitory control, involving extensive coordination between glutamatergic and GABAergic transmission, and the pathogenic loss of inhibitory control via impaired interneurons may contribute to aberrant gamma oscillations (13, 33, 114). Why then, do we observe both enhancements and reductions in gamma oscillatory power and coherence after cannabinoid exposure? Gamma signal is involved in maintaining local and global circuits, and a local disruption could lead to global changes in distal brain areas (13). Also, eCB tone is critical for coordinating neurodevelopment and early-life disruptions of the eCB, like adolescent cannabinoid exposure (115, 116), can lead to long-term baseline oscillatory changes implicated in the etiology of mental illnesses (26).

Although the review focused on schizophrenia, major depressive disorder, and bipolar disorder, it is important to consider which other serious mental illnesses may involve, or be precipitated or ameliorated by, cannabinoids and eCB modulation. Patients with attention-deficit/hyperactivity disorder, obsessive-compulsive disorder, anxiety-related disorders, and post-traumatic stress disorder all report greater rates of cannabis use and worsened outcomes (117, 118). Some of these patients may also benefit from therapeutic targeting of the eCB (119–123), highlighting the need for further research into this area.

Future studies should focus on comparing the effects of varying administration routes, toward a set of standardized methodologies used to examine cannabinoid exposure in models of mental illness, as these studies often use different routes and doses of cannabinoid administration which may contribute to inter-study variability. The other cannabinoids in the cannabis plant must also be investigated, as they demonstrate partial agonist activity for both CB1R and CB2R, and produce signature CB1R-dependent behavioral responses in mice tested using the cannabis tetrad (59). Additionally, whole-brain examinations must be completed to connect disparate observations of regional differences in brain activity both before, and after, acute and chronic cannabinoid exposure. Recent studies have used preclinical magnetic resonance imaging to examine the mechanisms underlying serious behavioral dysfunctions in some models of mental illness (34). Furthermore, differences in circuit activity may be related to different medications taken by patients, or inherent differences in brain functional connectivity and/or the measured state (124), which might make the reverse translation of these findings to animal models difficult. Thus, a holistic approach to studying and translating circuit dysfunctions in animal models is imperative. Finally, although the therapeutic relevance of disrupted oscillatory activity in cannabis use and serious mental illnesses is contested, modulation of gamma oscillations should be investigated to characterize the pathogenic nature of aberrant gamma signal, and to hopefully reveal a circuit-based mechanism that can be targeted for intervention (16, 39, 125).

## Author Contributions

BJ drafted the review and formatted Figures 1–4. JK formulated the idea for the review and guided the research and writing process. Both authors contributed to the article and approved the submitted version.

## Conflict of Interest

The authors declare that the research was conducted in the absence of any commercial or financial relationships that could be construed as a potential conflict of interest.

## References

[1] Lev-Ran S Le Foll B Mckenzie K George TP Rehm J. Cannabis use and cannabis use disorders among individuals with mental illness. Compr Psychiatry. (2013) 54:589–98. 10.1016/j.comppsych.2012.12.021 23375264

[2] Osuch E Vingilis E Ross E Forster C Summerhurst C. Cannabis use, addiction risk and functional impairment in youth seeking treatment for primary mood or anxiety concerns. Int J Adolesc Med Health. (2013) 25:309–14. 10.1515/ijamh-2013-0067 23839811

[3] Volkow ND. Substance use disorders in schizophrenia–clinical implications of comorbidity. Schizophr Bull. (2009) 35:469–72. 10.1093/schbul/sbp016 19325163 PMC2669586

[4] Koskinen J Lohonen J Koponen H Isohanni M Miettunen J. Rate of cannabis use disorders in clinical samples of patients with schizophrenia: a meta-analysis. Schizophr Bull. (2010) 36:1115–30. 10.1093/schbul/sbp031 19386576 PMC2963055

[5] Pinto JV Medeiros LS Santana Da Rosa G Santana De Oliveira CE Crippa JAS Passos IC. The prevalence and clinical correlates of cannabis use and cannabis use disorder among patients with bipolar disorder: a systematic review with meta-analysis and meta-regression. Neurosci Biobehav Rev. (2019) 101:78–84. 10.1016/j.neubiorev.2019.04.004 30974123

[6] Pacek LR Weinberger AH Zhu J Goodwin RD. Rapid increase in the prevalence of cannabis use among people with depression in the United States, 2005-17: the role of differentially changing risk perceptions. Addiction. (2020) 115:935–43. 10.1111/add.14883 31797462 PMC7156311

[7] Khokhar JY Dwiel LL Henricks AM Doucette WT Green AI. The link between schizophrenia and substance use disorder: a unifying hypothesis. Schizophr Res. (2018) 194:78–85. 10.1016/j.schres.2017.04.016 28416205 PMC6094954

[8] Drake RE Wallach MA. Dual diagnosis: 15 years of progress. Psychiatr Serv. (2000) 51:1126–9. 10.1176/appi.ps.51.9.1126 10970914

[9] Hunt GE Siegfried N Morley K Sitharthan T Cleary M. Psychosocial interventions for people with both severe mental illness and substance misuse. Schizophr Bull. (2014) 40:18–20. 10.1093/schbul/sbt160 24179148 PMC3885307

[10] Abush H Akirav I. Cannabinoids ameliorate impairments induced by chronic stress to synaptic plasticity and short-term memory. Neuropsychopharmacology. (2013) 38:1521–34. 10.1038/npp.2013.51 23426383 PMC3682147

[11] Aguilar DD Chen L Lodge DJ. Increasing endocannabinoid levels in the ventral pallidum restore aberrant dopamine neuron activity in the subchronic PCP rodent model of schizophrenia. Int J Neuropsychopharmacol. (2014) 18:pyu035. 10.1093/ijnp/pyu035 25539511 PMC4332795

[12] Aguilar DD Giuffrida A Lodge DJ. THC and endocannabinoids differentially regulate neuronal activity in the prefrontal cortex and hippocampus in the subchronic PCP model of schizophrenia. J Psychopharmacol. (2016) 30:169–81. 10.1177/0269881115612239 26510449 PMC5252830

[13] Atallah BV Scanziani M. Instantaneous modulation of gamma oscillation frequency by balancing excitation with inhibition. Neuron. (2009) 62:566–77. 10.1016/j.neuron.2009.04.027 19477157 PMC2702525

[14] Barz CS Bessaih T Abel T Feldmeyer D Contreras D. Sensory encoding in Neuregulin 1 mutants. Brain Struct Funct. (2016) 221:1067–81. 10.1007/s00429-014-0955-x 25515311 PMC5040327

[15] Dzirasa K Coque L Sidor MM Kumar S Dancy EA Takahashi JS. Lithium ameliorates nucleus accumbens phase-signaling dysfunction in a genetic mouse model of mania. J Neurosci. (2010) 30:16314–23. 10.1523/JNEUROSCI.4289-10.2010 21123577 PMC3165036

[16] Gazit T Friedman A Lax E Samuel M Zahut R Katz M. Programmed deep brain stimulation synchronizes VTA gamma band field potential and alleviates depressive-like behavior in rats. Neuropharmacology. (2015) 91:135–41. 10.1016/j.neuropharm.2014.12.003 25497452

[17] Goodwill HL Manzano-Nieves G Gallo M Lee HI Oyerinde E Serre T. Early life stress leads to sex differences in development of depressive-like outcomes in a mouse model. Neuropsychopharmacology. (2019) 44:711–20. 10.1038/s41386-018-0195-5 30188513 PMC6372611

[18] Hajos M Hoffmann WE Kocsis B. Activation of cannabinoid-1 receptors disrupts sensory gating and neuronal oscillation: relevance to schizophrenia. Biol Psychiatry. (2008) 63:1075–83. 10.1016/j.biopsych.2007.12.005 18261715

[19] Hudson R Renard J Norris C Rushlow WJ Laviolette SR. Cannabidiol counteracts the psychotropic side-effects of delta-9-tetrahydrocannabinol in the ventral hippocampus through bidirectional control of ERK1-2 phosphorylation. J Neurosci. (2019) 39:8762–77. 10.1523/JNEUROSCI.0708-19.2019 31570536 PMC6820200

[20] Iniguez SD Riggs LM Nieto SJ Dayrit G Zamora NN Shawhan KL. Social defeat stress induces a depression-like phenotype in adolescent male c57BL/6 mice. Stress. (2014) 17:247–55. 10.3109/10253890.2014.910650 24689732 PMC5534169

[21] Iturra-Mena AM Aguilar-Rivera M Arriagada-Solimano M Perez-Valenzuela C Fuentealba P Dagnino-Subiabre A. Impact of stress on gamma oscillations in the rat nucleus accumbens during spontaneous social interaction. Front Behav Neurosci. (2019) 13:151. 10.3389/fnbeh.2019.00151 31354444 PMC6636240

[22] Khalid A Kim BS Seo BA Lee ST Jung KH Chu K. Gamma oscillation in functional brain networks is involved in the spontaneous remission of depressive behavior induced by chronic restraint stress in mice. BMC Neurosci. (2016) 17:4. 10.1186/s12868-016-0239-x 26759057 PMC4710024

[23] Lecca S Luchicchi A Scherma M Fadda P Muntoni AL Pistis M. Delta(9)-tetrahydrocannabinol during adolescence attenuates disruption of dopamine function induced in rats by maternal immune activation. Front Behav Neurosci. (2019) 13:202. 10.3389/fnbeh.2019.00202 31551729 PMC6743372

[24] Lee H Dvorak D Fenton AA. Targeting neural synchrony deficits is sufficient to improve cognition in a schizophrenia-related neurodevelopmental model. Front Psychiatry. (2014) 5:15. 10.3389/fpsyt.2014.00015 24592242 PMC3924579

[25] Linge R Jimenez-Sanchez L Campa L Pilar-Cuellar F Vidal R Pazos A. Cannabidiol induces rapid-acting antidepressant-like effects and enhances cortical 5-HT/glutamate neurotransmission: role of 5-HT1A receptors. Neuropharmacology. (2016) 103:16–26. 10.1016/j.neuropharm.2015.12.017 26711860

[26] Moussa-Tooks AB Larson ER Gimeno AF Leishman E Bartolomeo LA Bradshaw HB. Long-term aberrations to cerebellar endocannabinoids induced by early-life stress. Sci Rep. (2020) 10:7236. 10.1038/s41598-020-64075-4 32350298 PMC7190863

[27] Nelong TF Jenkins BW Perreault ML Khokhar JY. Extended attenuation of corticostriatal power and coherence after acute exposure to vapourized Δ9-tetrahydrocannabinol in rats. Can J Addict. (2019) 10:60–6. 10.1097/CXA.0000000000000063 32944610 PMC7494223

[28] Nguyen JD Aarde SM Vandewater SA Grant Y Stouffer DG Parsons LH. Inhaled delivery of Delta(9)-tetrahydrocannabinol (THC) to rats by e-cigarette vapor technology. Neuropharmacology. (2016) 109:112–20. 10.1016/j.neuropharm.2016.05.021 27256501 PMC4970926

[29] Park H Rhee J Lee S Chung C. Selectively impaired endocannabinoid-dependent long-term depression in the lateral habenula in an animal model of depression. Cell Rep. (2017) 20:289–96. 10.1016/j.celrep.2017.06.049 28700932

[30] Narayanan B O'neil K Berwise C Stevens MC Calhoun VD Clementz BA. Resting state electroencephalogram oscillatory abnormalities in schizophrenia and psychotic bipolar patients and their relatives from the bipolar and schizophrenia network on intermediate phenotypes study. Biol Psychiatry. (2014) 76:456–65. 10.1016/j.biopsych.2013.12.008 24439302 PMC5045030

[31] Renard J Loureiro M Rosen LG Zunder J De Oliveira C Schmid S. Cannabidiol counteracts amphetamine-induced neuronal and behavioral sensitization of the mesolimbic dopamine pathway through a novel mTOR/p70S6 kinase signaling pathway. J Neurosci. (2016) 36:5160–9. 10.1523/JNEUROSCI.3387-15.2016 27147666 PMC4854973

[32] Renard J Szkudlarek HJ Kramar CP Jobson CEL Moura K Rushlow WJ. Adolescent THC exposure causes enduring prefrontal cortical disruption of GABAergic inhibition and dysregulation of sub-cortical dopamine function. Sci Rep. (2017) 7:11420. 10.1038/s41598-017-11645-8 28900286 PMC5595795

[33] Sauer JF Struber M Bartos M. Impaired fast-spiking interneuron function in a genetic mouse model of depression. Elife. (2015) 4:e04979. 10.7554/eLife.04979.028 25735038 PMC4374525

[34] Seewoo BJ Hennessy LA Feindel KW Etherington SJ Croarkin PE Rodger J. Validation of chronic restraint stress model in young adult rats for the study of depression using longitudinal multimodal MR imaging. eNeuro. (2020) 7:ENEURO.0113-20.2020. 10.1523/ENEURO.0113-20.2020 32669346 PMC7396811

[35] Segev A Rubin AS Abush H Richter-Levin G Akirav I. Cannabinoid receptor activation prevents the effects of chronic mild stress on emotional learning and LTP in a rat model of depression. Neuropsychopharmacology. (2014) 39:919–33. 10.1038/npp.2013.292 24141570 PMC3924526

[36] Seillier A Martinez AA Giuffrida A. Differential effects of Delta9-tetrahydrocannabinol dosing on correlates of schizophrenia in the sub-chronic PCP rat model. PLoS ONE. (2020) 15:e0230238. 10.1371/journal.pone.0230238 32163506 PMC7067407

[37] Sigurdsson T Stark KL Karayiorgou M Gogos JA Gordon JA. Impaired hippocampal-prefrontal synchrony in a genetic mouse model of schizophrenia. Nature. (2010) 464:763–7. 10.1038/nature08855 20360742 PMC2864584

[38] Taffe MA Creehan KM Vandewater SA Kerr TM Cole M Effects of Δ9-tetrahydrocannabinol (THC) vapor inhalation in Sprague-Dawley and Wistar rats. Exp Clin Psychopharmacol. (2020). 10.1037/pha0000373. [Epub ahead of print]. PMC8376092 32297788

[39] Tchenio A Lecca S Valentinova K Mameli M. Limiting habenular hyperactivity ameliorates maternal separation-driven depressive-like symptoms. Nat Commun. (2017) 8:1135. 10.1038/s41467-017-01192-1 29074844 PMC5658350

[40] Valvassori SS Elias G De Souza B Petronilho F Dal-Pizzol F Kapczinski F. Effects of cannabidiol on amphetamine-induced oxidative stress generation in an animal model of mania. J Psychopharmacol. (2011) 25:274–80. 10.1177/0269881109106925 19939866

[41] Voget M Rummel J Avchalumov Y Sohr R Haumesser JK Rea E. Altered local field potential activity and serotonergic neurotransmission are further characteristics of the Flinders sensitive line rat model of depression. Behav Brain Res. (2015) 291:299–305. 10.1016/j.bbr.2015.05.027 26025511

[42] Young AM Stubbendorff C Valencia M Gerdjikov TV. Disruption of medial prefrontal synchrony in the subchronic phencyclidine model of schizophrenia in rats. Neuroscience. (2015) 287:157–63. 10.1016/j.neuroscience.2014.12.014 25542422 PMC4317768

[43] Cortes-Briones J Skosnik PD Mathalon D Cahill J Pittman B Williams A. Delta9-THC disrupts gamma (gamma)-band neural oscillations in humans. Neuropsychopharmacology. (2015) 40:2124–34. 10.1038/npp.2015.53 25709097 PMC4613601

[44] Skosnik PD Hajos M Cortes-Briones JA Edwards CR Pittman BP Hoffmann WE. Cannabinoid receptor-mediated disruption of sensory gating and neural oscillations: a translational study in rats and humans. Neuropharmacology. (2018) 135:412–23. 10.1016/j.neuropharm.2018.03.036 29604295 PMC6091633

[45] Kamarajan C Porjesz B Jones KA Choi K Chorlian DB Padmanabhapillai A. The role of brain oscillations as functional correlates of cognitive systems: a study of frontal inhibitory control in alcoholism. Int J Psychophysiol. (2004) 51:155–80. 10.1016/j.ijpsycho.2003.09.004 14693365 PMC3766846

[46] Uhlhaas PJ Singer W. Abnormal neural oscillations and synchrony in schizophrenia. Nat Rev Neurosci. (2010) 11:100–13. 10.1038/nrn2774 20087360

[47] Başar E Brain oscillations in neuropsychiatric disease. Dial Clin Neurosci. (2013) 15:291–300. 10.31887/DCNS.2013.15.3/ebasar PMC3811101 24174901

[48] Sherif MA Cortes-Briones JA Ranganathan M Skosnik PD. Cannabinoid-glutamate interactions and neural oscillations: implications for psychosis. Eur J Neurosci. (2018) 48:2890–902. 10.1111/ejn.13800 29247465

[49] Whittington MA Traub RD Adams NE. A future for neuronal oscillation research. Brain Neurosci Adv. (2018) 2:2398212818794827. 10.1177/2398212818794827 32166146 PMC7058255

[50] Steigerwald S Wong PO Cohen BE Ishida JH Vali M Madden E. Smoking, vaping, and use of edibles and other forms of marijuana among U.S. Adults. Ann Intern Med. (2018) 169:890–2. 10.7326/M18-1681 30167665 PMC6296858

[51] Jung KM Piomelli D Cannabinoids and Endocannabinoids. In: Pfaff D Volkow N editors. Neuroscience in the 21st Century. New York, NY: Springer (2016). 10.1007/978-1-4939-3474-4_136

[52] García-Gutiérrez MS Manzanares J. Overexpression of CB2 cannabinoid receptors decreased vulnerability to anxiety and impaired anxiolytic action of alprazolam in mice. J Psychopharmacol. (2010) 25:111–20. 10.1177/0269881110379507 20837564

[53] Bahi A Al Mansouri S Al Memari E Al Ameri M Nurulain SM Ojha S. β-Caryophyllene, a CB2 receptor agonist produces multiple behavioral changes relevant to anxiety and depression in mice. Physiol Behav. (2014) 135:119–24. 10.1016/j.physbeh.2014.06.003 24930711

[54] Li Y Kim J. Distinct roles of neuronal and microglial CB2 cannabinoid receptors in the mouse hippocampus. Neuroscience. (2017) 363:11–25. 10.1016/j.neuroscience.2017.08.053 28888955

[55] Cécyre B Bachand I Papineau F Brochu C Casanova C Bouchard J-F. Cannabinoids affect the mouse visual acuity via the cannabinoid receptor type 2. Sci Rep. (2020) 10:15819. 10.1038/s41598-020-72553-y 32978469 PMC7519129

[56] Bambico FR Katz N Debonnel G Gobbi G. Cannabinoids elicit antidepressant-like behavior and activate serotonergic neurons through the medial prefrontal cortex. J Neurosci. (2007) 27:11700–11. 10.1523/JNEUROSCI.1636-07.2007 17959812 PMC6673235

[57] De Petrocellis L Ligresti A Moriello AS Allara M Bisogno T Petrosino S. Effects of cannabinoids and cannabinoid-enriched Cannabis extracts on TRP channels and endocannabinoid metabolic enzymes. Br J Pharmacol. (2011) 163:1479–94. 10.1111/j.1476-5381.2010.01166.x 21175579 PMC3165957

[58] De Mello Schier AR De Oliveira Ribeiro NP Coutinho DS Machado S Arias-Carrión O Crippa JA. Antidepressant-like and anxiolytic-like effects of cannabidiol: a chemical compound of Cannabis sativa. CNS Neurol Disord Drug Targets. (2014) 13:953–60. 10.2174/1871527313666140612114838 24923339

[59] Zagzoog A Mohamed KA Kim HJJ Kim ED Frank CS Black T. *In vitro* and *in vivo* pharmacological activity of minor cannabinoids isolated from *Cannabis sativa*. Sci Rep. (2020) 10:20405. 10.1038/s41598-020-77175-y 33230154 PMC7684313

[60] Mclaughlin RJ. Toward a translationally relevant preclinical model of cannabis use. Neuropsychopharmacology. (2018) 43:213. 10.1038/npp.2017.191 29192672 PMC5719100

[61] Mccutcheon RA Reis Marques T Howes OD. Schizophrenia-an overview. JAMA Psychiatry. (2019) 77:201–10. 10.1001/jamapsychiatry.2019.3360 31664453

[62] Powell CM Miyakawa T. Schizophrenia-relevant behavioral testing in rodent models: a uniquely human disorder? Biol Psychiatry. (2006) 59:1198–207. 10.1016/j.biopsych,.2006.05.008 16797265 PMC3928106

[63] Brady AM. The Neonatal Ventral Hippocampal Lesion (NVHL) rodent model of schizophrenia. Curr Protoc Neurosci. (2016) 77:51–9. 10.1002/cpns.15 27696361 PMC5113298

[64] Jaaro-Peled H Gene models of schizophrenia: DISC1 mouse models. In: Akira S editor. Genetic Models of Schizophrenia. (Amsterdam, Netherlands: Elsevier) (2009) p. 75–86. 10.1016/S0079-6123(09)17909-8 20302820

[65] Tomoda T Sumitomo A Jaaro-Peled H Sawa A. Utility and validity of DISC1 mouse models in biological psychiatry. Neuroscience. (2016) 321:99–107. 10.1016/j.neuroscience.2015.12.061 26768401 PMC4803604

[66] Mei L Xiong WC. Neuregulin 1 in neural development, synaptic plasticity and schizophrenia. Nat Rev Neurosci. (2008) 9:437–52. 10.1038/nrn2392 18478032 PMC2682371

[67] Haddad FL Patel SV Schmid S. Maternal immune activation by poly I:C as a preclinical model for neurodevelopmental disorders: a focus on autism and schizophrenia. Neurosci Biobehav Rev. (2020) 113:546–67. 10.1016/j.neubiorev.2020.04.012 32320814

[68] Tseng KY Chambers RA Lipska BK. The neonatal ventral hippocampal lesion as a heuristic neurodevelopmental model of schizophrenia. Behav Brain Res. (2009) 204:295–305. 10.1016/j.bbr.2008.11.039 19100784 PMC2735579

[69] Khokhar JY Todd TP. Behavioral predictors of alcohol drinking in a neurodevelopmental rat model of schizophrenia and co-occurring alcohol use disorder. Schizophr Res. (2018) 194:91–7. 10.1016/j.schres.2017.02.029 28285734 PMC5591749

[70] Reynolds GP Neill JC. Modelling the cognitive and neuropathological features of schizophrenia with phencyclidine. J Psychopharmacol. (2016) 30:1141–4. 10.1177/0269881116667668 27624147

[71] W.H.O Depression. Available online at: https://www.who.int/news-room/fact-sheets/detail/depression (2020).

[72] Will CC Aird F Redei EE. Selectively bred Wistar-Kyoto rats: an animal model of depression and hyper-responsiveness to antidepressants. Mol Psychiatry. (2003) 8:925–32. 10.1038/sj.mp.4001345 14593430

[73] Overstreet DH Wegener G. The flinders sensitive line rat model of depression−25 years and still producing. Pharmacol Rev. (2013) 65:143–55. 10.1124/pr.111.005397 23319547

[74] Aleksandrova LR Wang YT Phillips AG. Evaluation of the Wistar-Kyoto rat model of depression and the role of synaptic plasticity in depression and antidepressant response. Neurosci Biobehav Rev. (2019) 105:1–23. 10.1016/j.neubiorev.2019.07.007 31336112

[75] Willner P Gruca P Lason M Tota-Glowczyk K Litwa E Niemczyk M. Validation of chronic mild stress in the Wistar-Kyoto rat as an animal model of treatment-resistant depression. Behav Pharmacol. (2019) 30:239–50. 10.1097/FBP.0000000000000431 30204592

[76] Gururajan A Reif A Cryan JF Slattery DA. The future of rodent models in depression research. Nat Rev Neurosci. (2019) 20:686–701. 10.1038/s41583-019-0221-6 31578460

[77] Schmidt MV Wang XD Meijer OC. Early life stress paradigms in rodents: potential animal models of depression? Psychopharmacology. (2011) 214:131–40. 10.1007/s00213-010-2096-0 21086114

[78] Soderlund J Lindskog M. Relevance of rodent models of depression in clinical practice: can we overcome the obstacles in translational neuropsychiatry? Int J Neuropsychopharmacol. (2018) 21:668–76. 10.1093/ijnp/pyy037 29688411 PMC6030948

[79] Valverde O. Participation of the cannabinoid system in the regulation of emotional-like behaviour. Curr Pharm Des. (2005) 11:3421–9. 10.2174/138161205774370780 16250845

[80] Valverde O Torrens M. CB1 receptor-deficient mice as a model for depression. Neuroscience. (2012) 204:193–206. 10.1016/j.neuroscience.2011.09.031 21964469

[81] Shen CJ Zheng D Li KX Yang JM Pan HQ Yu XD. Cannabinoid CB1 receptors in the amygdalar cholecystokinin glutamatergic afferents to nucleus accumbens modulate depressive-like behavior. Nat Med. (2019) 25:337–49. 10.1038/s41591-018-0299-9 30643290

[82] Martin M Ledent C Parmentier M Maldonado R Valverde O. Involvement of CB1 cannabinoid receptors in emotional behaviour. Psychopharmacology. (2002) 159:379–87. 10.1007/s00213-001-0946-5 11823890

[83] N.I.M.H Bipolar Disorder. Available online at: https://www.nimh.nih.gov/health/topics/bipolar-disorder/index.shtml (accessed August 10, 2020) (2020).

[84] Cosgrove VE Kelsoe JR Suppes T. Toward a valid animal model of bipolar disorder: how the research domain criteria help bridge the clinical-basic science divide. Biol Psychiatry. (2016) 79:62–70. 10.1016/j.biopsych.2015.09.002 26531027

[85] Kristensen M Nierenberg AA Ostergaard SD. Face and predictive validity of the ClockDelta19 mouse as an animal model for bipolar disorder: a systematic review. Mol Psychiatry. (2018) 23:70–80. 10.1038/mp.2017.192 29112195

[86] Wu N Cepeda C Zhuang X Levine MS. Altered corticostriatal neurotransmission and modulation in dopamine transporter knock-down mice. J Neurophysiol. (2007) 98:423–32. 10.1152/jn.00971.2006 17522168

[87] Young JW Goey AK Minassian A Perry W Paulus MP Geyer MA. The mania-like exploratory profile in genetic dopamine transporter mouse models is diminished in a familiar environment and reinstated by subthreshold psychostimulant administration. Pharmacol Biochem Behav. (2010) 96:7–15. 10.1016/j.pbb.2010.03.014 20363246 PMC2878916

[88] Young JW Van Enkhuizen J Winstanley CA Geyer MA. Increased risk-taking behavior in dopamine transporter knockdown mice: further support for a mouse model of mania. J Psychopharmacol. (2011) 25:934–43. 10.1177/0269881111400646 21421642 PMC3568506

[89] Van Enkhuizen J Henry BL Minassian A Perry W Milienne-Petiot M Higa KK. Reduced dopamine transporter functioning induces high-reward risk-preference consistent with bipolar disorder. Neuropsychopharmacology. (2014) 39:3112–22. 10.1038/npp.2014.170 25005251 PMC4229584

[90] Kwiatkowski MA Hellemann G Sugar CA Cope ZA Minassian A Perry W. Dopamine transporter knockdown mice in the behavioral pattern monitor: a robust, reproducible model for mania-relevant behaviors. Pharmacol Biochem Behav. (2019) 178:42–50. 10.1016/j.pbb.2017.12.007 29289701 PMC10014035

[91] Bossé R Fumagalli F Jaber M Giros B Gainetdinov RR Wetsel WC. Anterior pituitary hypoplasia and dwarfism in mice lacking the dopamine transporter. Neuron. (1997) 19:127–38. 10.1016/S0896-6273(00)80353-0 9247269

[92] Dzirasa K Ramsey AJ Takahashi DY Stapleton J Potes JM Williams JK. Hyperdopaminergia and NMDA receptor hypofunction disrupt neural phase signaling. J Neurosci. (2009) 29:8215–24. 10.1523/JNEUROSCI.1773-09.2009 19553461 PMC2731697

[93] Cho RY Konecky RO Carter CS. Impairments in frontal cortical gamma synchrony and cognitive control in schizophrenia. Proc Natl Acad Sci USA. (2006) 103:19878–83. 10.1073/pnas.0609440103 17170134 PMC1750867

[94] Uhlhaas PJ Linden DE Singer W Haenschel C Lindner M Maurer K. Dysfunctional long-range coordination of neural activity during Gestalt perception in schizophrenia. J Neurosci. (2006) 26:8168–75. 10.1523/JNEUROSCI.2002-06.2006 16885230 PMC6673788

[95] Haenschel C Bittner RA Waltz J Haertling F Wibral M Singer W. Cortical oscillatory activity is critical for working memory as revealed by deficits in early-onset schizophrenia. J Neurosci. (2009) 29:9481–9. 10.1523/JNEUROSCI.1428-09.2009 19641111 PMC6666530

[96] Ruggiero RN Rossignoli MT De Ross JB Hallak JEC Leite JP Bueno-Junior LS. Cannabinoids and vanilloids in schizophrenia: neurophysiological evidence and directions for basic research. Front Pharmacol. (2017) 8:399. 10.3389/fphar.2017.00399 28680405 PMC5478733

[97] Liao SC Wu CT Huang HC Cheng WT Liu YH. Major depression detection from EEG signals using kernel eigen-filter-bank common spatial patterns. Sensors. (2017) 17:1385. 10.3390/s17061385 28613237 PMC5492453

[98] Fitzgerald PJ Watson BO. Gamma oscillations as a biomarker for major depression: an emerging topic. Transl Psychiatry. (2018) 8:177. 10.1038/s41398-018-0239-y 30181587 PMC6123432

[99] O'donnell BF Hetrick WP Vohs JL Krishnan GP Carroll CA Shekhar A. Neural synchronization deficits to auditory stimulation in bipolar disorder. Neuroreport. (2004) 15:1369–72. 10.1097/01.wnr.0000127348.64681.b2 15167568

[100] Rass O Krishnan G Brenner CA Hetrick WP Merrill CC Shekhar A. Auditory steady state response in bipolar disorder: relation to clinical state, cognitive performance, medication status, and substance disorders. Bipolar Disord. (2010) 12:793–803. 10.1111/j.1399-5618.2010.00871.x 21176026 PMC3060563

[101] Basar E Guntekin B Atagun I Turp Golbasi B Tulay E Ozerdem A. Brain's alpha activity is highly reduced in euthymic bipolar disorder patients. Cogn Neurodyn. (2012) 6:11–20. 10.1007/s11571-011-9172-y 23372616 PMC3253163

[102] Oda Y Onitsuka T Tsuchimoto R Hirano S Oribe N Ueno T. Gamma band neural synchronization deficits for auditory steady state responses in bipolar disorder patients. PLoS ONE. (2012) 7:e39955. 10.1371/journal.pone.0039955 22792199 PMC3390322

[103] Kam JW Bolbecker AR O'donnell BF Hetrick WP Brenner CA. Resting state EEG power and coherence abnormalities in bipolar disorder and schizophrenia. J Psychiatr Res. (2013) 47:1893–901. 10.1016/j.jpsychires.2013.09.009 24090715 PMC4015517

[104] Raver SM Keller A. Permanent suppression of cortical oscillations in mice after adolescent exposure to cannabinoids: receptor mechanisms. Neuropharmacology. (2014) 86:161–73. 10.1016/j.neuropharm.2014.07.006 25036610 PMC4188721

[105] Murray RM Englund A Abi-Dargham A Lewis DA Di Forti M Davies C. Cannabis-associated psychosis: neural substrate and clinical impact. Neuropharmacology. (2017) 124:89–104. 10.1016/j.neuropharm.2017.06.018 28634109

[106] D'souza DC Perry E Macdougall L Ammerman Y Cooper T Wu YT. The psychotomimetic effects of intravenous delta-9-tetrahydrocannabinol in healthy individuals: implications for psychosis. Neuropsychopharmacology. (2004) 29:1558–72. 10.1038/sj.npp.1300496 15173844

[107] Cortes-Briones JA Cahill JD Skosnik PD Mathalon DH Williams A Sewell RA. The psychosis-like effects of Delta(9)-tetrahydrocannabinol are associated with increased cortical noise in healthy humans. Biol Psychiatry. (2015) 78:805–13. 10.1016/j.biopsych.2015.03.023 25913109 PMC4627857

[108] Nottage JF Stone J Murray RM Sumich A Bramon-Bosch E Ffytche D. Delta-9-tetrahydrocannabinol, neural oscillations above 20 Hz and induced acute psychosis. Psychopharmacology. (2015) 232:519–28. 10.1007/s00213-014-3684-1 25038870 PMC4302232

[109] Kedzior KK Martin-Iverson MT. Chronic cannabis use is associated with attention-modulated reduction in prepulse inhibition of the startle reflex in healthy humans. J Psychopharmacol. (2006) 20:471–84. 10.1177/0269881105057516 16174673

[110] Edwards CR Skosnik PD Steinmetz AB O'donnell BF Hetrick WP. Sensory gating impairments in heavy cannabis users are associated with altered neural oscillations. Behav Neurosci. (2009) 123:894–904. 10.1037/a0016328 19634950 PMC3536882

[111] Solowij N Broyd SJ Beale C Prick JA Greenwood LM Van Hell H. Therapeutic effects of prolonged cannabidiol treatment on psychological symptoms and cognitive function in regular cannabis users: a pragmatic open-label clinical trial. Cannabis Cannabinoid Res. (2018) 3:21–34. 10.1089/can.2017.0043 29607408 PMC5870061

[112] Zheng K An JJ Yang F Xu W Xu ZQ Wu J. TrkB signaling in parvalbumin-positive interneurons is critical for gamma-band network synchronization in hippocampus. Proc Natl Acad Sci USA. (2011) 108:17201–6. 10.1073/pnas.1114241108 21949401 PMC3193255

[113] Singh F Shu IW Hsu SH Link P Pineda JA Granholm E. Modulation of frontal gamma oscillations improves working memory in schizophrenia. Neuroimage Clin. (2020) 27:102339. 10.1016/j.nicl.2020.102339 32712452 PMC7390812

[114] Lee Y Zhang Y Kim S Han K. Excitatory and inhibitory synaptic dysfunction in mania: an emerging hypothesis from animal model studies. Exp Mol Med. (2018) 50:12. 10.1038/s12276-018-0028-y 29628501 PMC5938027

[115] Thorpe HHA Hamidullah S Jenkins BW Khokhar JY. Adolescent neurodevelopment and substance use: receptor expression and behavioral consequences. Pharmacol Ther. (2019) 206:107431. 10.1016/j.pharmthera.2019.107431 31706976

[116] Hamidullah S Lutelmowski CD Creighton SD Luciani KR Frie JA Winters BD. Effects of vapourized THC and voluntary alcohol drinking during adolescence on cognition, reward, and anxiety-like behaviours in rats. Prog Neuro Psychopharmacol Biol Psychiatry. (2020) 106:110141. 10.1016/j.pnpbp.2020.110141 33069816

[117] Toftdahl NG Nordentoft M Hjorthoj C. Prevalence of substance use disorders in psychiatric patients: a nationwide Danish population-based study. Soc Psychiatry Psychiatr Epidemiol. (2016) 51:129–40. 10.1007/s00127-015-1104-4 26260950

[118] Anker E Haavik J Heir T. Alcohol and drug use disorders in adult attention-deficit/hyperactivity disorder: prevalence and associations with attention-deficit/hyperactivity disorder symptom severity and emotional dysregulation. World J Psychiatry. (2020) 10:202–11. 10.5498/wjp.v10.i9.202 33014721 PMC7515748

[119] Patel S Hillard CJ. Role of endocannabinoid signaling in anxiety and depression. Curr Top Behav Neurosci. (2009) 1:347–71. 10.1007/978-3-540-88955-7_14 21104391 PMC3808114

[120] Trezza V Campolongo P. The endocannabinoid system as a possible target to treat both the cognitive and emotional features of post-traumatic stress disorder (PTSD). Front Behav Neurosci. (2013) 7:100. 10.3389/fnbeh.2013.00100 23950739 PMC3739026

[121] Blessing EM Steenkamp MM Manzanares J Marmar CR. Cannabidiol as a potential treatment for anxiety disorders. Neurotherapeutics. (2015) 12:825–36. 10.1007/s13311-015-0387-1 26341731 PMC4604171

[122] Cohen J Wei Z Phang J Laprairie RB Zhang Y. Cannabinoids as an emerging therapy for posttraumatic stress disorder and substance use disorders. J Clin Neurophysiol. (2020) 37:28–34. 10.1097/wnp.0000000000000612 31895187

[123] Kayser RR Haney M Raskin M Arout C Simpson HB. Acute effects of cannabinoids on symptoms of obsessive-compulsive disorder: A human laboratory study. Depress Anxiety. (2020). 37:801–11. 10.1002/da.23032 32383271 PMC7423713

[124] Atagun MI. Brain oscillations in bipolar disorder and lithium-induced changes. Neuropsychiatr Dis Treat. (2016) 12:589–601. 10.2147/NDT.S100597 27022264 PMC4788370

[125] Stasiulewicz A Znajdek K Grudzien M Pawinski T Sulkowska AJI. A guide to targeting the endocannabinoid system in drug design. Int J Mol Sci. (2020) 21. 10.3390/ijms21082778 32316328 PMC7216112

[126] Swanson L. W. (2018). Brain maps 4.0-structure of the rat brain: an open access atlas with global nervous system nomenclature ontology and flatmaps. J Comp Neurol 526, 935–943. 10.1002/cne.24381 29277900 PMC5851017

